# Recurrent spontaneous pneumothorax during pregnancy: a case report

**DOI:** 10.1186/1752-1947-3-81

**Published:** 2009-10-21

**Authors:** Preeti Jain, Kavita Goswami

**Affiliations:** 1 Department of Obstetrics and Gynaecology, University Hospital Coventry and Warwickshire NHS Trust, Coventry, CV2 2DX, UK

## Abstract

**Introduction:**

Spontaneous recurrent pneumothorax during pregnancy is a rare condition. Few cases have been reported previously in the literature. There is no universal guideline for the management of this condition. Treatment options include conservative management with intercostal drain and surgical management in the form of thoracotomy or video-assisted thoracoscopy.

**Case presentation:**

We report a case of recurrent spontaneous pneumothorax in a 38-year-old Afro-Caribbean woman on her third trimester of pregnancy. The disease was managed with the insertion of an intercostal drain on three occasions, which was then followed by surgical intervention immediately after pregnancy.

**Conclusion:**

The diagnosis of pneumothorax should be considered in the differential diagnosis of pregnant women experiencing chest pain and dyspnoea. No adverse maternal or foetal outcome has been reported in well-managed cases. Management involves good coordination between the obstetric and surgical teams.

## Introduction

Spontaneous recurrent pneumothorax is an unusual condition and its exact incidence is unknown. Due to physiological changes during pregnancy, any condition leading to impairment in ventilation is poorly tolerated.

This condition is related to the presence of small apical blebs or bullae in the absence of a known significant pulmonary disease [1]. Prompt and accurate diagnosis is crucial as sudden respiratory comprise can occur secondary to tension pneumothorax [2]. The diagnosis of pneumothorax should be excluded in any pregnant women experiencing chest pain and dyspnoea.

## Case presentation

**Figure 1:**
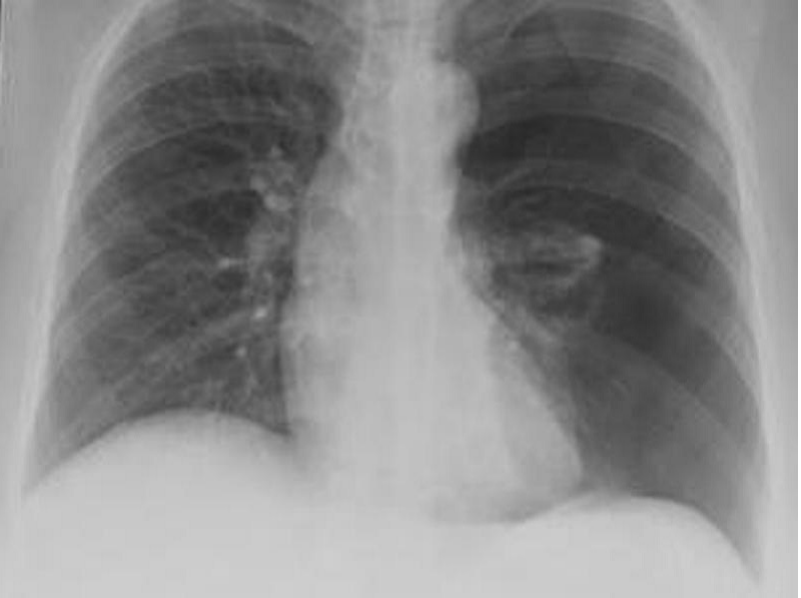
**Chest X-ray showing large left pneumothorax with collapsed lung**.

A 38-year-old Afro-Caribbean woman presented at 32 weeks of pregnancy with chest pain and shortness of breath. She was a non-smoker with no history of pulmonary disease. Her body mass index (BMI) was 38 and she had conceived following *in vitro *fertilisation (IVF) treatment. Infection screen gave negative results. On examination, she was found to be mildly tachypnoeic with decreased air entry and had a hyperresonant percussion note over the left hemithorax. Pulse oximetry showed an oxygen saturation of 95% on air. Blood gas analysis confirmed normal arterial oxygen and carbon dioxide tension. Chest X-ray revealed a large left pneumothorax with a collapsed lung (Figure 1).

On examination and ultrasound scan, uterine fibroids were diagnosed with the largest one measuring 22 cm. She had problems with fibroid degeneration and dental abscess during this particular pregnancy.

**Figure 2:**
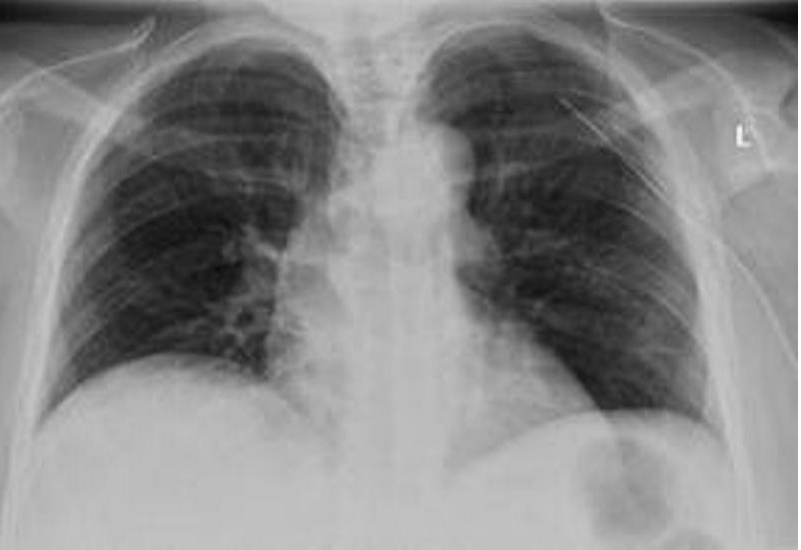
**Resolved pneumothorax after chest drain insertion**.

Pneumothorax was treated using chest tube drain, and it resolved completely in a few days (Figure 2).

She was readmitted with another pneumothorax two weeks later and on the 34^th ^week of gestation. A chest drain was reinserted and removed three days later following resolution of the pneumothorax.

**Figure 3:**
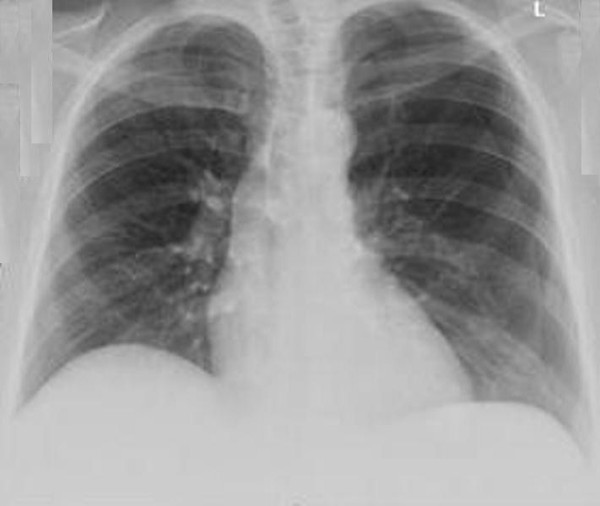
**Recurrence of left pneumothorax three weeks after the patient's initial presentation**.

Unfortunately, the pneumothorax recurred after another three weeks and a chest drain was again inserted (Figure 3).

She had also started developing pre-eclampsia when she was nearly 37 weeks in her pregnancy. Labour was induced at this stage. She had an epidural during labour. She had normal vaginal delivery with the baby born in good condition and weighing around 2760 gm.

The chest drain was removed after delivery, and this was followed by video-assisted thoracoscopic surgery (VATS) and apical pleurectomy.

The postoperative period was uneventful and she made a smooth recovery.

## Discussion

Spontaneous pneumothorax during pregnancy is a rare pathological condition where air collects into the pleural cavity accompanied by lung collapse without any trauma to the lung or chest wall. This condition is generally caused by the rupture of small apical blebs or bullae in the absence of other significant pulmonary disease. Common risk factors noted are an underlying respiratory infection, asthma or a prior history of pneumothorax [3].

Only a few cases have been reported in the literature. The exact incidence of the disease is unknown. Most of the patients present with chest pain and dyspnoea, which is similar to what our patient experienced. The main risk for the mother is respiratory compromise while foetal risks include a reduction in oxygen supply and preterm labour.

Treatment is based on the magnitude of pneumothorax. The risk of recurrence is 30% to 40%, particularly during labour [3].

The management of recurrent pneumothorax in pregnant women should be no different from that in non-pregnant women. Up to 75% of patients are treated with chest tube drainage as a first line of treatment. Surgery is an option for persistent or recurrent pneumothorax despite adequate drainage. Surgery could be either via thoracotomy or VATS, which is commonly done through pleurectomy or mechanical scrubbing of the pleural surface [4]. The optimal time for the surgical intervention is during the second trimester.

To prevent an increase in the intrathoracic pressure during pushing stage, epidural analgesia and instrumental delivery to cut short the second stage are recommended for those who have not undergone definitive surgical management [1].

Vaginal delivery has been the most common method of delivery in patients that are pregnant. With our patient, the situation was further complicated by the presence of massive uterine fibroids and a pregnancy complication of pre-eclampsia. The options were a planned Caesarean section with uterine artery catheterisation or a prostaglandin induction of labour. The latter was successful with a short second stage and a normal vaginal delivery.

## Conclusion

Recurrent pneumothorax during pregnancy can be treated in the same way as in non-pregnant women. Prognosis is generally good for both the mother and the baby. Thoracotomy and VATS have been increasingly successful as procedures for managing patients. There were no maternal or foetal complications reported in those who underwent antepartum surgical intervention.

## Abbreviations

BMI: body mass index; IVF: *in vitro *fertilisation; VATS: video-assisted thoracoscopic surgery.

## Competing interests

The authors declare that they have no competing interests.

## Authors' contributions

PJ wrote the case report & literature review sections of the manuscript. KG secured the patient's consent to publish this case report. She also did the final revision of the manuscript. Both authors were involved in managing the patient.

## Consent

Written informed consent was obtained from the patient for publication of this case report and any accompanying images. A copy of the written consent is available for review by the Editor-in-chief of this journal.

## References

[B1] Van Winter JT Nicholas FC Pairolero PC Ney JA Ogburn PL Jr Management of spontaneous pneumothorax during pregnancy: case report and review of the literature Mayo clinic proceedings 1996 71 249 252 10.4065/71.3.249 8594282

[B2] Sills ES Meinecke HM Dixson GR Johnson AM Management approach for recurrent spontaneous pneumothorax in consecutive pregnancies based on clinical and radiographic findings J Cardiothoracic Surg 2006 1 35 10.1186/1749-8090-1-35 PMC1624824 17052345

[B3] Dhalla SS Teskey JM Surgical management of recurrent spontaneous pneumothorax during pregnancy Chest 1985 86 301 302 10.1378/chest.88.2.301 4017687

[B4] Wong MK Leung WC Wang JK Lao TT Ip MS Lam WK Ho JC Recurrent pneumothorax in pregnancy: what should we do after placing an intercostal drain? Hong Kong Med J 2006 12 375 380 17028358

